# Supplementary material for: Northern preference for terrestrial electromagnetic energy input from space weather
Source: Nat Commun. 2021 Jan 8;12:199. doi: 10.1038/s41467-020-20450-3 (PMC7794368; doi:10.1038/s41467-020-20450-3)
Supplement: Supplementary file 1 — Supplementary Information [file 41467_2020_20450_MOESM1_ESM.pdf]

Supplementary Information for

Northern Preference for Terrestrial Electromagnetic Energy Input from Space Weather

I. P. Pakhotin*[1], I. R. Mann[1], K. Xie[1], J. Burchill[2], D. Knudsen[2]

[1] Department of Physics, University of Alberta, Edmonton, Alberta, Canada

[2] University of Calgary, Calgary, Alberta, Canada

Contents of this file

Supplementary Figures 1-10

*contact email: pakhotin@ualberta.ca

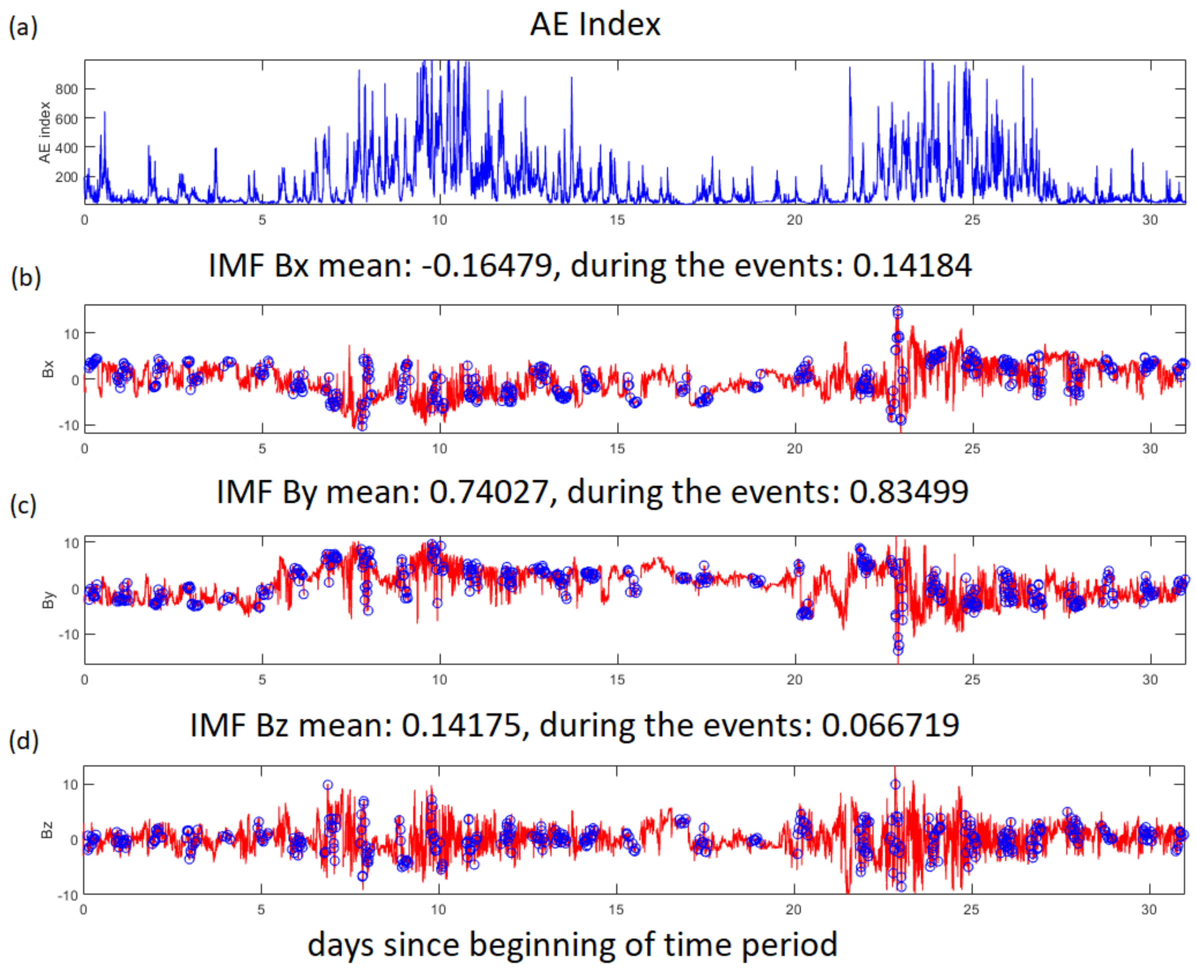

Supplementary Figure 1: Characteristics of the AE index and IMF for the 15 November-15 December 2016 period. Panel (a) shows the time series of the Auroral Electrojet (AE) index (b) IMF Bx, (c) IMF By, (d) IMF Bz, all from OMNIWeb, with the times of Swarm events included in the analysis being marked on (b), (c) and (d) with blue circles for the corresponding IMF orientations.

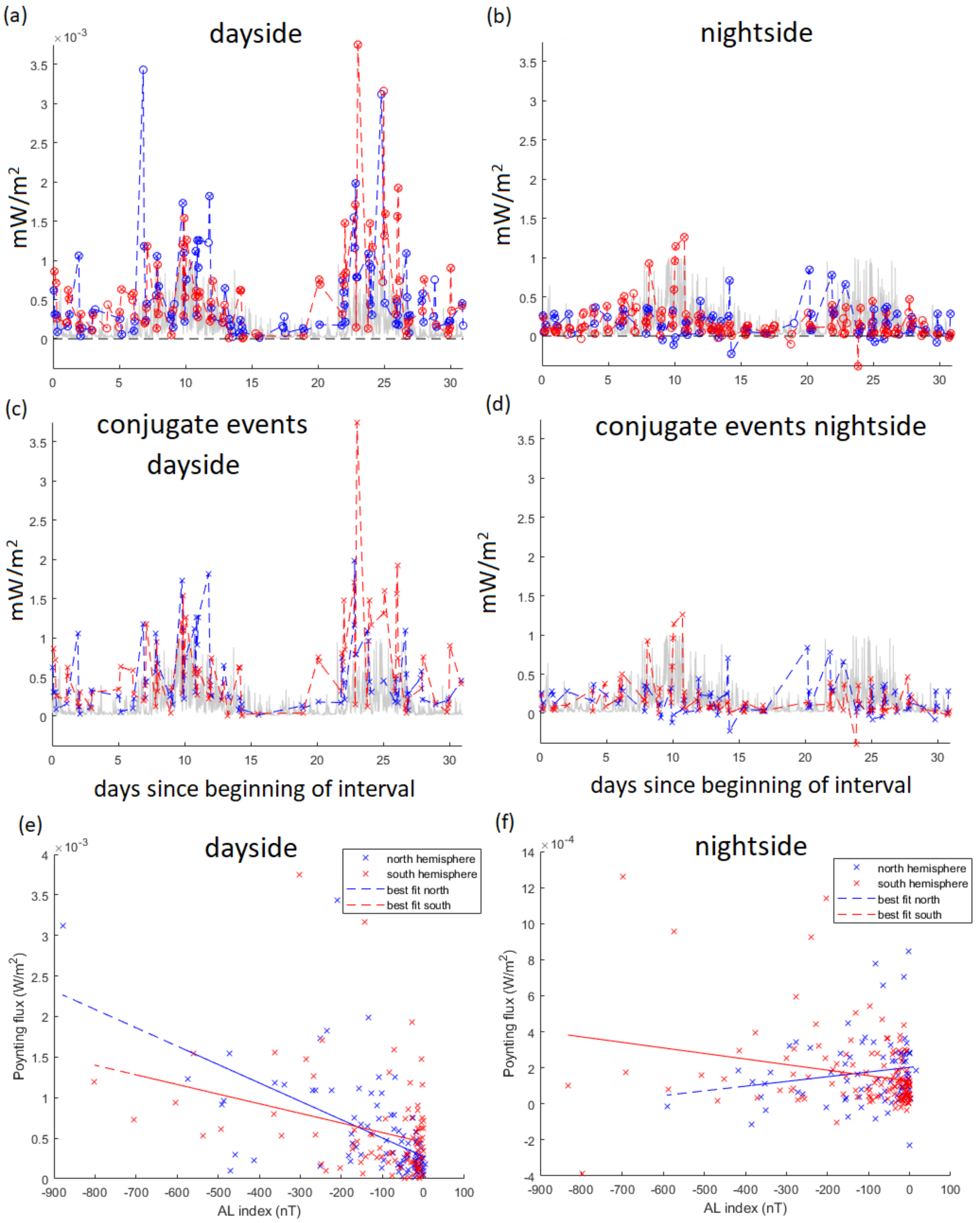

Supplementary Figure 2: Poynting flux event plots for the 15 November-15 December 2016 period. Panel (a) shows the Poynting fluxes for dayside events (marked with circles) for the northern and southern hemispheres (blue and red, respectively); dashed lines represent linear connections between individual events. Panel (b) shows the same as (a) for nightside. Conjugate events in the statistical event

datasets, defined as those which occurred on conjugate hemispheres within 30 minutes of each other, are marked with crosses. These conjugate events are isolated and plotted in panel (c) for dayside and (d) for nightside. In panels (a) to (d) the AE Index from Supplementary Figure 1a is plotted in the background in light gray to give context to the variability of Poynting fluxes in response to varying solar wind and geomagnetic driving conditions. Finally, (e) and (f) show scatter plots of the AL index vs Poynting flux for the two hemispheres for the dayside and nightside respectively, along with lines of best fit.

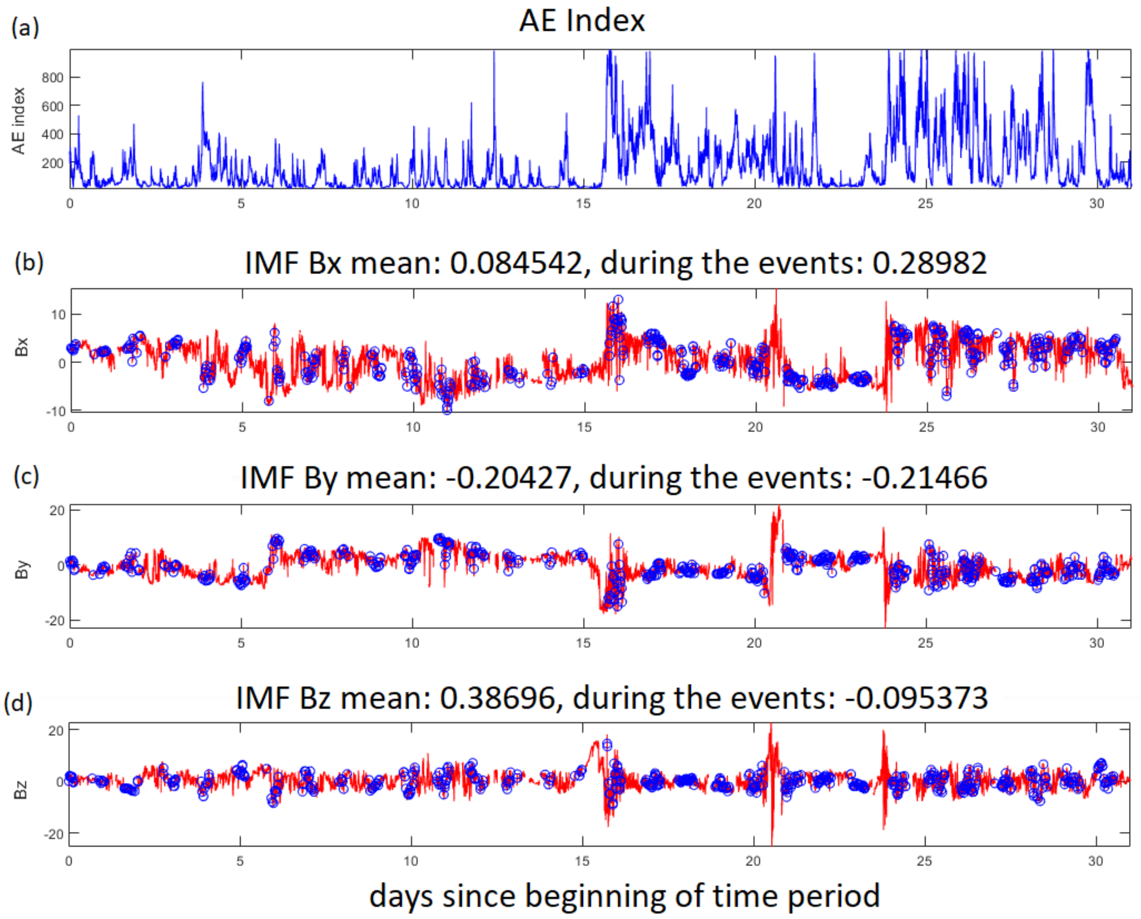

Supplementary Figure 3: Characteristics of the AE index and IMF for the 20 February-25 March, 2016 period (same format as in Supplementary Figure 1). Panel (a) shows the time series of the Auroral Electrojet (AE) index (b) IMF Bx, (c) IMF By, (d) IMF Bz, all from OMNIWeb, with the times of Swarm events included in the analysis being marked on (b), (c) and (d) with blue circles for the corresponding IMF orientations.

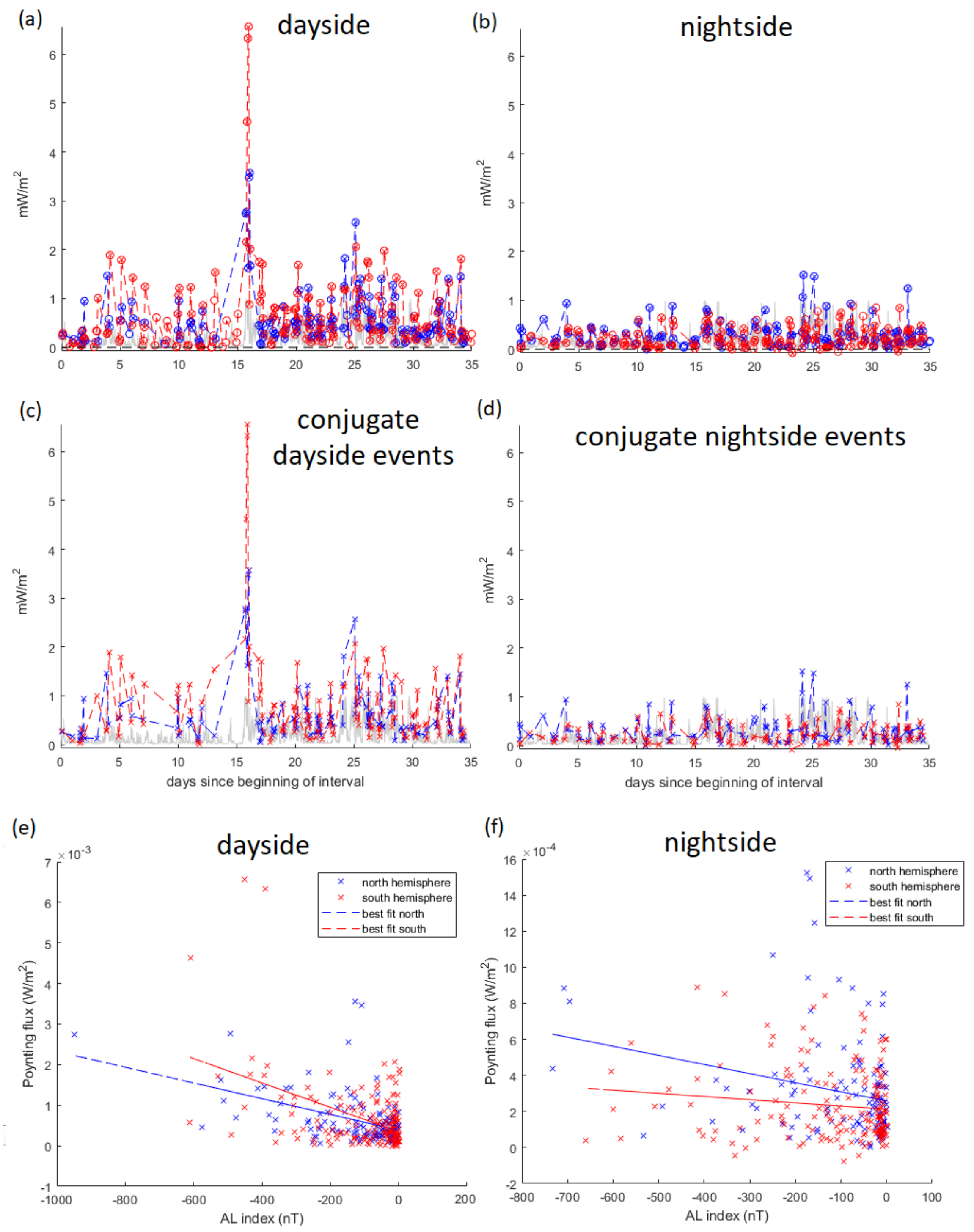

Supplementary Figure 4: Poynting flux event plots for the 20 February-25 March, 2016 period (same format as in Supplementary Figure 2). Panel (a) shows the Poynting fluxes for dayside events (marked with circles) for the northern and southern hemispheres (blue and red, respectively); dashed lines represent linear connections between individual events. Panel (b) shows the same as (a) for nightside. Conjugate events in the statistical event datasets, defined as those which occurred on conjugate hemispheres within 30 minutes of each other, are marked with crosses. These conjugate events are

isolated and plotted in panel (c) for dayside and (d) for nightside. In panels (a) to (d) the AE Index from Supplementary Figure 3a is plotted in the background in light gray to give context to the variability of Poynting fluxes in response to varying solar wind and geomagnetic driving conditions. Finally, (e) and (f) show scatter plots of the AL index vs Poynting flux for the two hemispheres for the dayside and nightside respectively, along with lines of best fit.

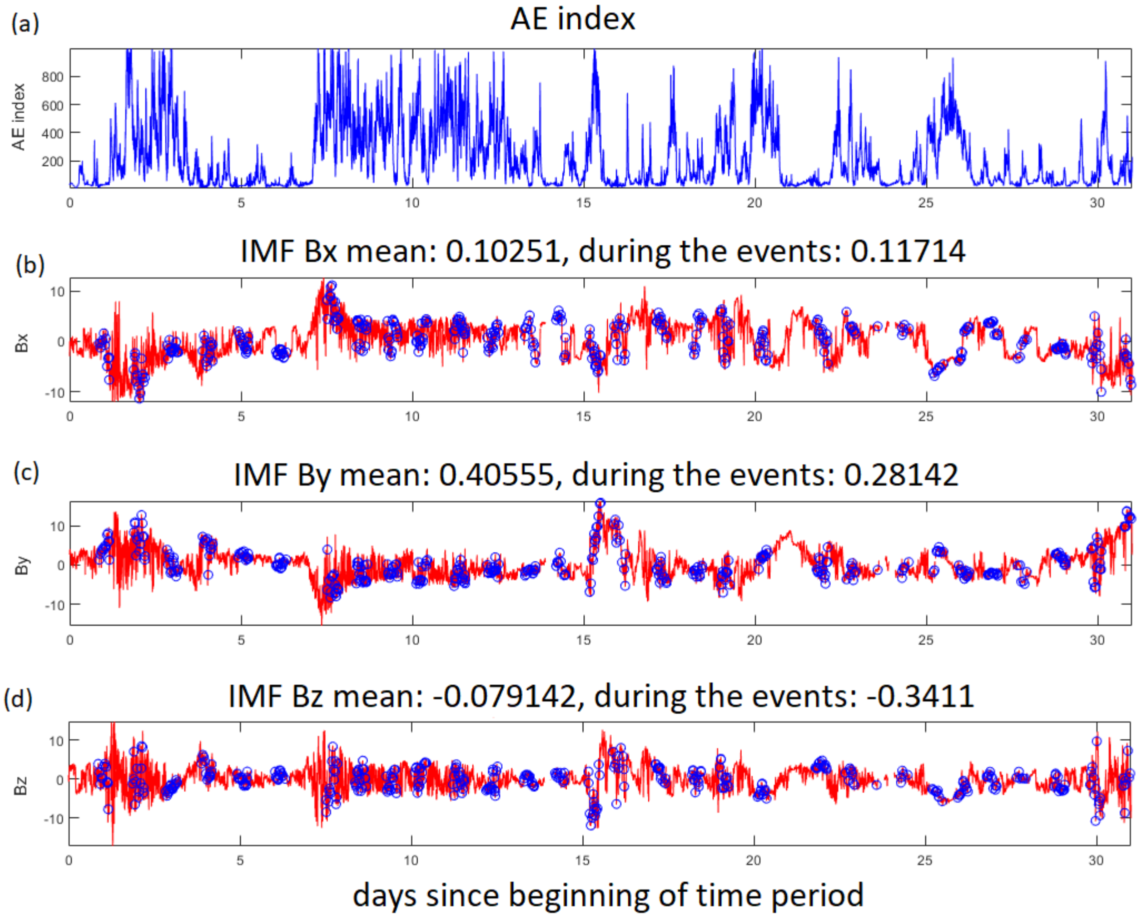

Supplementary Figure 5: Characteristics of the AE index and IMF for the 20 March-20 April, 2017 time period (same format as in Supplementary Figure 1). Panel (a) shows the time series of the Auroral Electrojet (AE) index (b) IMF Bx, (c) IMF By, (d) IMF Bz, all from OMNIWeb, with the times of Swarm events included in the analysis being marked on (b), (c) and (d) with blue circles for the corresponding IMF orientations.

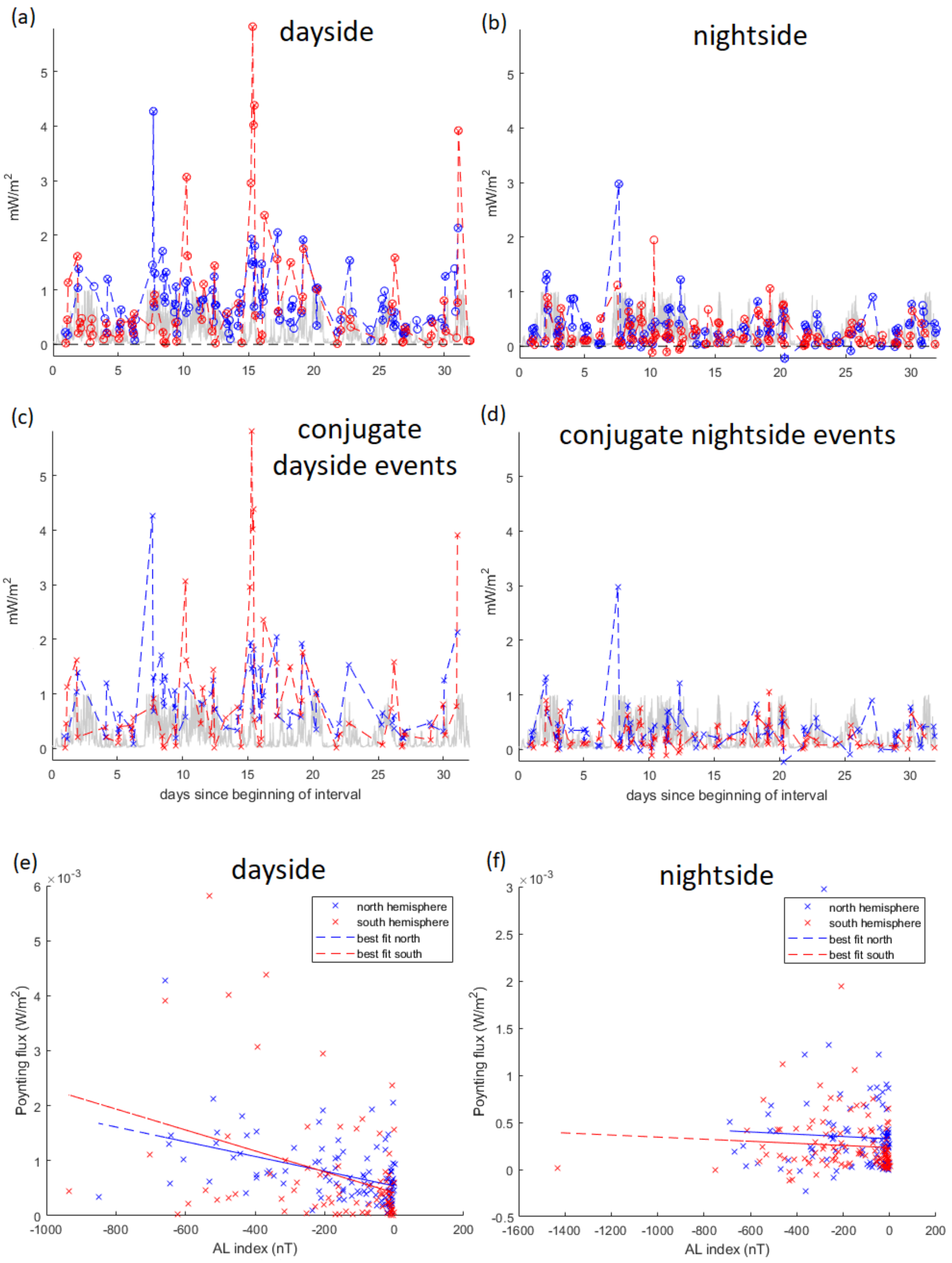

Supplementary Figure 6: Poynting flux event plots for the 20 March-20 April, 2017 period (same format as in Supplementary Figure 2). Panel (a) shows the Poynting fluxes for dayside events (marked with circles) for the northern and southern hemispheres (blue and red, respectively); dashed lines represent linear connections between individual events. Panel (b) shows the same as (a) for nightside. Conjugate

events in the statistical event datasets, defined as those which occurred on conjugate hemispheres within 30 minutes of each other, are marked with crosses. These conjugate events are isolated and plotted in panel (c) for dayside and (d) for nightside. In panels (a) to (d) the AE Index from Supplementary Figure 5a is plotted in the background in light gray to give context to the variability of Poynting fluxes in response to varying solar wind and geomagnetic driving conditions. Finally, (e) and (f) show scatter plots of the AL index vs Poynting flux for the two hemispheres for the dayside and nightside respectively, along with lines of best fit.

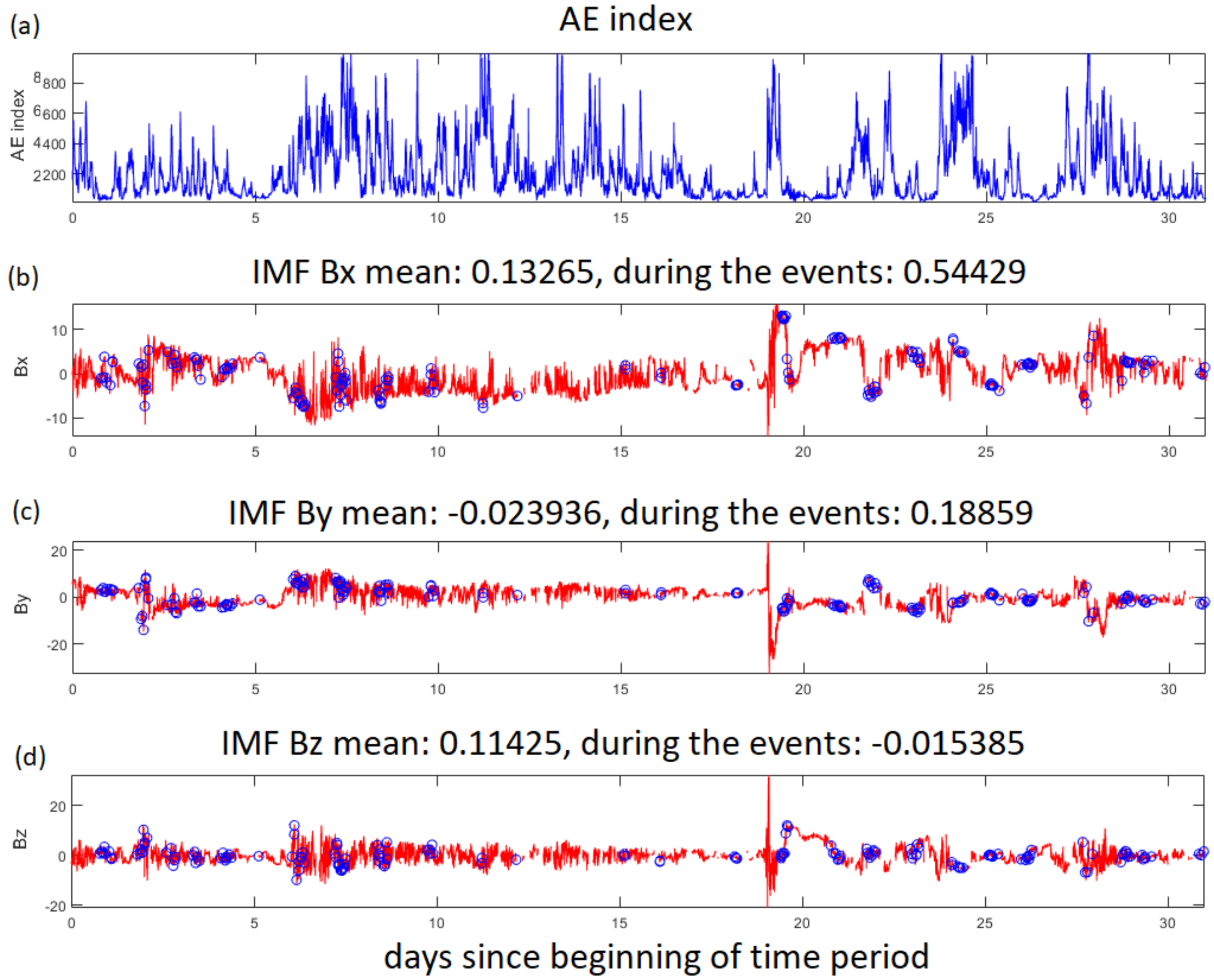

Supplementary Figure 7: Characteristics of the AE index and IMF for the 1-31 July, 2016 time period (same format as in Supplementary Figure 1). Panel (a) shows the time series of the Auroral Electrojet (AE) index (b) IMF Bx, (c) IMF By, (d) IMF Bz, all from OMNIWeb, with the times of Swarm events included in the analysis being marked on (b), (c) and (d) with blue circles for the corresponding IMF orientations.

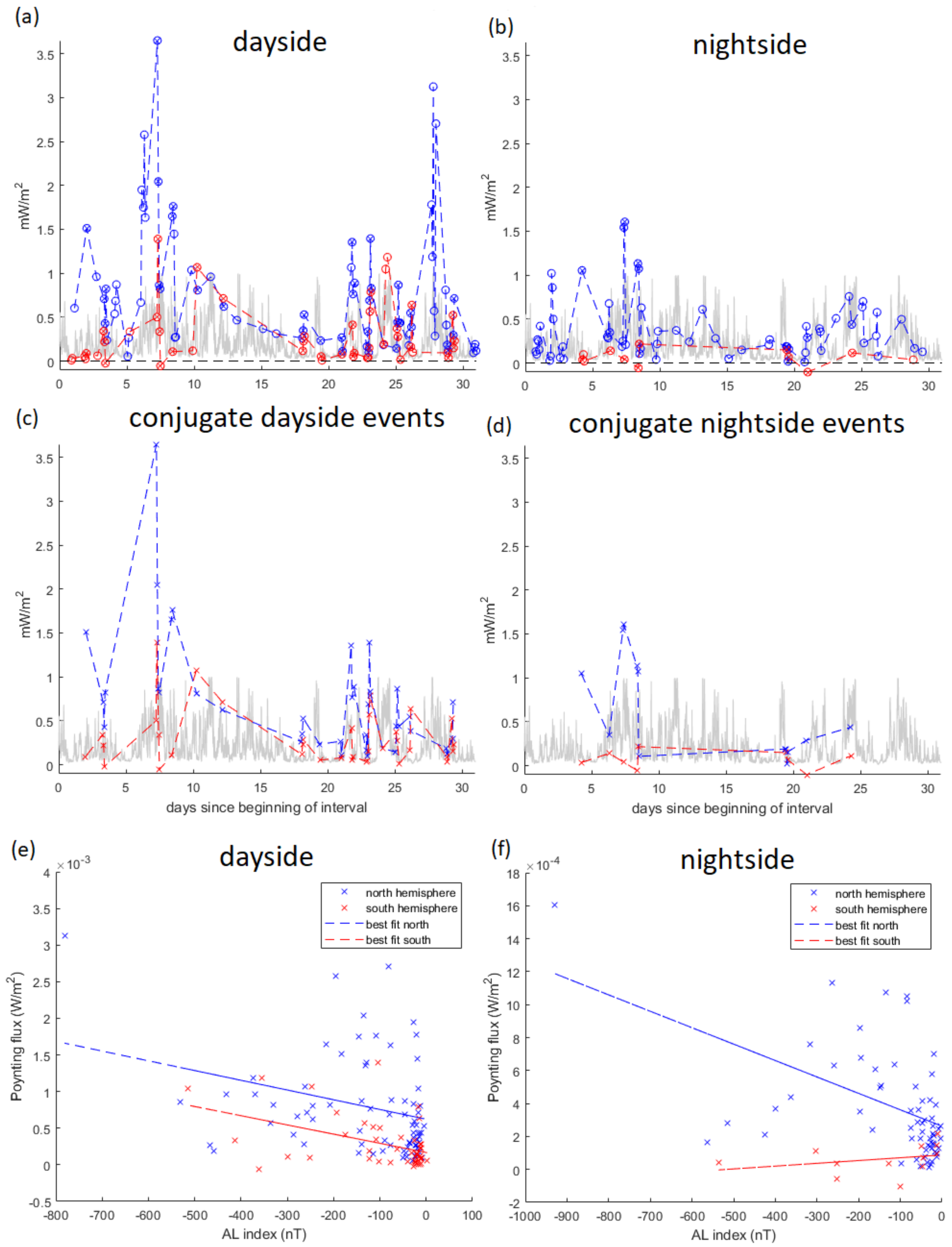

Supplementary Figure 8: Poynting flux event plots for the 1-31 July, 2016 period (same format as in Supplementary Figure 2). Panel (a) shows the Poynting fluxes for dayside events (marked with circles) for the northern and southern hemispheres (blue and red, respectively); dashed lines represent linear

connections between individual events. Panel (b) shows the same as (a) for nightside. Conjugate events in the statistical event datasets, defined as those which occurred on conjugate hemispheres within 30 minutes of each other, are marked with crosses. These conjugate events are isolated and plotted in panel (c) for dayside and (d) for nightside. In panels (a) to (d) the AE Index from Supplementary Figure 7a is plotted in the background in light gray to give context to the variability of Poynting fluxes in response to varying solar wind and geomagnetic driving conditions. Finally, (e) and (f) show scatter plots of the AL index vs Poynting flux for the two hemispheres for the dayside and nightside respectively, along with lines of best fit.

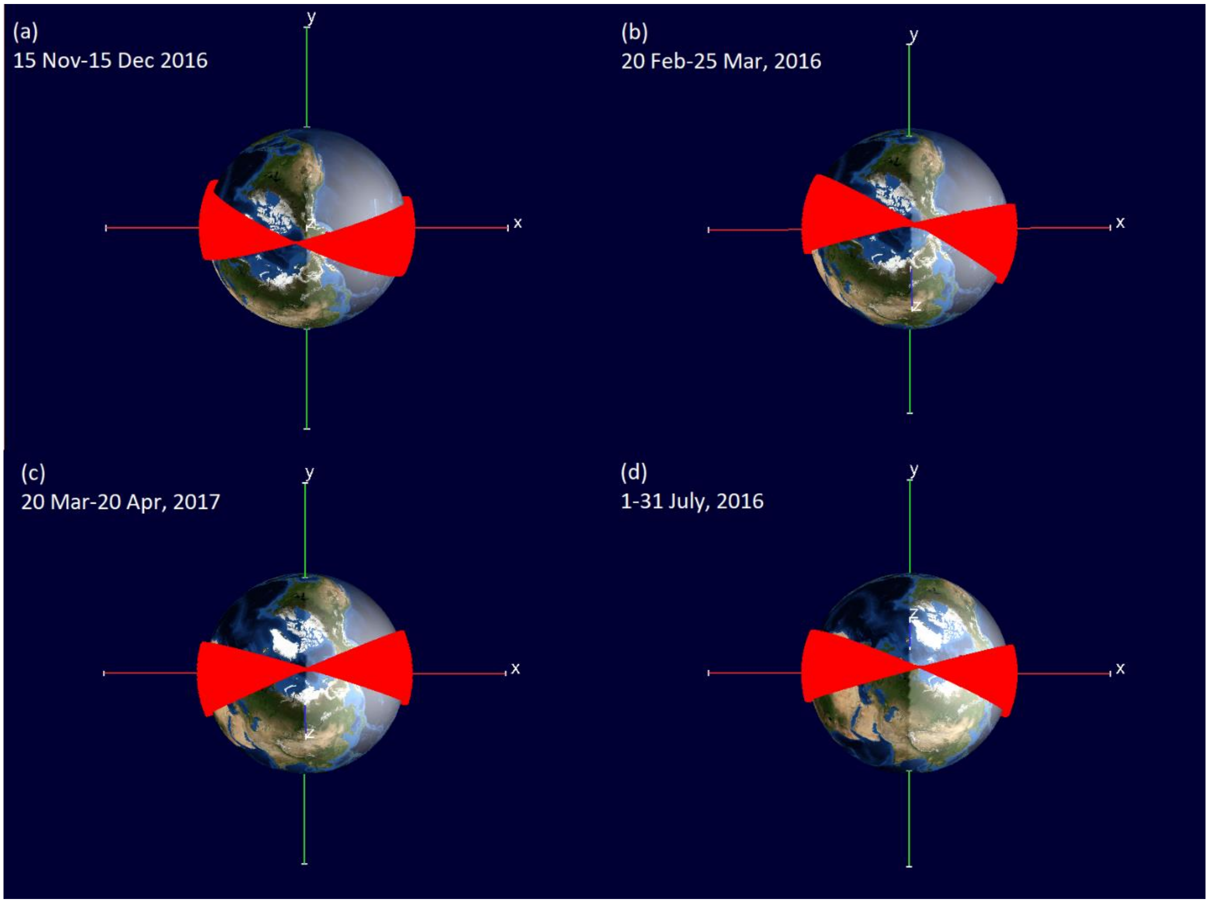

Supplementary Figure 9: NASA Satellite Situation Center (SSC) 4D Orbit Viewer figure of the Swarm A satellite orbit for the four seasonal time periods presented in Supplementary Figures 1-4, showing the noon-midnight orientation of the orbit plane for all examined seasons: (a) shows the 15 Nov-15 Dec 2016 period, (b) shows the 20 Feb-25 March 2016 period, (c) shows the 20 March-20 April, 2017 period, and (d) shows the 1-31 July, 2016 period.

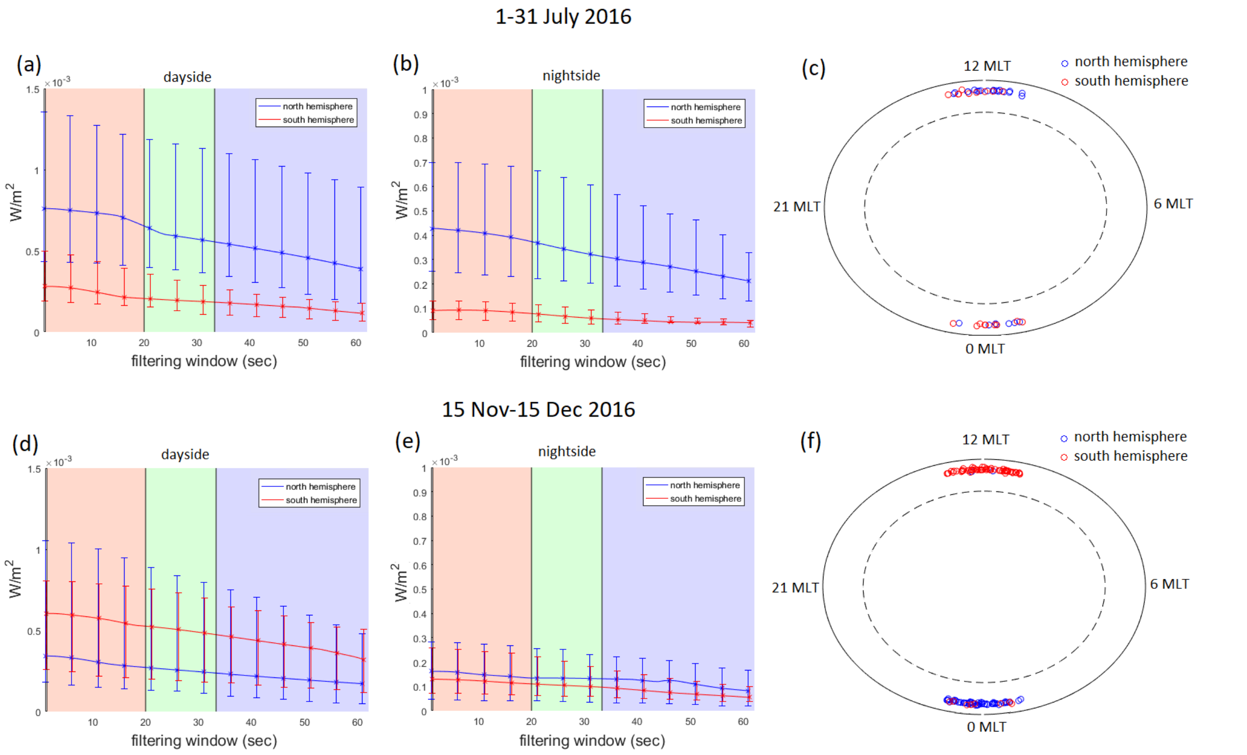

Supplementary Figure 10: Same as Figure 1 for one month of noon-midnight orbits during northern near-summer solstice (Panels a-c) and northern near-winter solstice (Panels d-f), but limited to local times of auroral zone passes of only one hour either side of the 12 MLT and 0 MLT sectors. The plots (a, b, d, e) show the median Poynting flux power values (solid line) and the 25% and 75% quartiles (error bars). Panels (a), (b), (d) and (e) use the same analysis as (a)-(d) in Figure 1, while Panels (c) and (f) show the occurrence distributions of individual events on a MLAT vs MLT map. The same characteristic northern preference in Figure 1 is also verified here.
